# Combined Hydrophobicity and Mechanical Durability through Surface Nanoengineering

**DOI:** 10.1038/srep09260

**Published:** 2015-04-08

**Authors:** Paul R. Elliott, Stephen P. Stagon, Hanchen Huang, David U. Furrer, Sergei F. Burlatsky, Thomas P. Filburn

**Affiliations:** 1 Department of Mechanical Engineering, University of Connecticut, Storrs, Connecticut 06268, USA; 2 Department of Mechanical Engineering, University of North Florida, Jacksonville, Florida 32224, USA; 3 Department of Mechanical and Industrial Engineering, Northeastern University, Boston, Massachusetts 02115, USA; 4 Pratt & Whitney, 1 Financial Plaza, East Hartford, Connecticut 06103, USA; 5 United Technologies Research Center, 411 Silver Ln, East Hartford, Connecticut 06118, USA; 6 Department of Mechanical Engineering, University of Hartford, West Hartford, Connecticut 06117, USA

## Abstract

This paper reports combined hydrophobicity and mechanical durability through the nanoscale engineering of surfaces in the form of nanorod-polymer composites. Specifically, the hydrophobicity derives from nanoscale features of mechanically hard ZnO nanorods and the mechanical durability derives from the composite structure of a hard ZnO nanorod core and soft polymer shell. Experimental characterization correlates the morphology of the nanoengineered surfaces with the combined hydrophobicity and mechanical durability, and reveals the responsible mechanisms. Such surfaces may find use in applications, such as boat hulls, that benefit from hydrophobicity and require mechanical durability.

Inspired by the hydrophobic self-cleaning lotus leaf in nature, humans have pursued synthetic hydrophobic surfaces for decades^1^,^2^. In an effort to mimic the surface of the lotus leaf, micro and nanoscale design has led to a range of structured surfaces that are hydrophobic^3^,^4^,^5^. The combination of hydrophobicity and mechanical durability is rarely found in nature. While the surface of the lotus leaf is hydrophobic, it is not subject to mechanical abrasion and its mechanical durability is not an issue. However, in technological applications, such as self-cleaning paints and low drag boat hulls^2^,^5^, surfaces are often subjected to mechanical abrasion, and mechanical durability is critically important.

Before proposing and developing new surfaces, it is beneficial to analyze the advantages and disadvantages of existing approaches. Lanthanide oxides form an intrinsically hydrophobic surface that is resistant to high temperatures and abrasive wear, but such materials are prohibitively expensive for large-scale technological applications due to the scarcity of rare earth materials^6^. Carbon nanotubes in an epoxy spray exhibit hydrophobicity and a low change in surface roughness after wear testing. However, wetting properties after mechanical wear are inconclusive^7^. Further, carbon nanotubes remain expensive and difficult to make in large quantities. Silica micro- and nano-particles in an epoxy spray coating have been used to achieve super-hydrophobicity with a low change in surface roughness after wear testing, but wetting properties after mechanical wear are not reported^8^.

The wetting properties determine the contact angle of water on a surface. The static contact angle, θ_s_, is the angle between the water-air interface and the water-solid interface, at the solid-liquid-air interface of a horizontal surface. In contrast, for the dynamic contact angle, it is different at the front and the back of a droplet moving along an incline^9^. Hereafter, contact angle refers to the static contact angle, unless specified otherwise. A surface is hydrophobic (θ_s_ > 90°) when it bonds weakly with water, or hydrophilic (θ_s_ < 90°) when it bonds strongly with water. In addition to the intrinsic surface chemical properties, surface geometrical roughness also affects the contact angle. As water comes into contact with the entire rough surface, the roughness makes a hydrophobic surface even more hydrophobic and a hydrophilic surface even more hydrophilic, as described by the Wenzel model^10^. However, when air is trapped between the water and a rough surface, the effective water-solid interface area is reduced, so the surface becomes more hydrophobic, as described by the Cassie-Baxter model^11^. The degree of air trapping offers a mechanism of controlling hydrophobicity.

In this paper, we design the degree of air trapping for hydrophobicity control, and further design a zinc oxide (ZnO)-polymer composite for mechanical durability. Here, the ZnO is mechanically hard and the polymer is mechanically resistant to wear, more so than the ZnO nanorods. By coating the ZnO nanorods, we take advantage of the combined strength of ZnO and toughness of polymer as in typical ceramic-polymer composites^12^. Here, we choose ZnO nanorods due to their easy and inexpensive syntheses. Additionally, the unique photonic properties of ZnO give it special applicability in light absorbing and emitting devices. ZnO nanorods with polymer shells have been shown to be applicable in photovoltaic cells^13^, and light emitting diodes^14^. The coupling of these properties and mechanical toughness may be beneficial for emerging technologies. Further, we choose polyurethane as the polymer because it is resistant to outdoor environment, has the right viscosity for desirable thickness of film and coating on the nanorods, and has a wetting angle of about 90° for both hydrophilic and hydrophobic control.

Figure 1 schematically illustrates the concept of hydrophobicity control. First, one may control the spacing, Ls, and the diameter of nanorods, D, to achieve superhydrophobic (θ_s_ > 150°) surfaces^9^. Second, one may control the effective height, Δ, through the polymer thickness, Hp, and the nanorod height, Hr. For hydrophobicity, Δ must be sufficiently large – larger than the water depth B, so air trapping is possible^15^,^16^. On the other hand, mechanical durability may require that Δ is not too large. This paper focuses on the easily controllable Δ to demonstrate the control of hydrophobicity, as well as mechanical durability.

## Results

**Figure 2:**
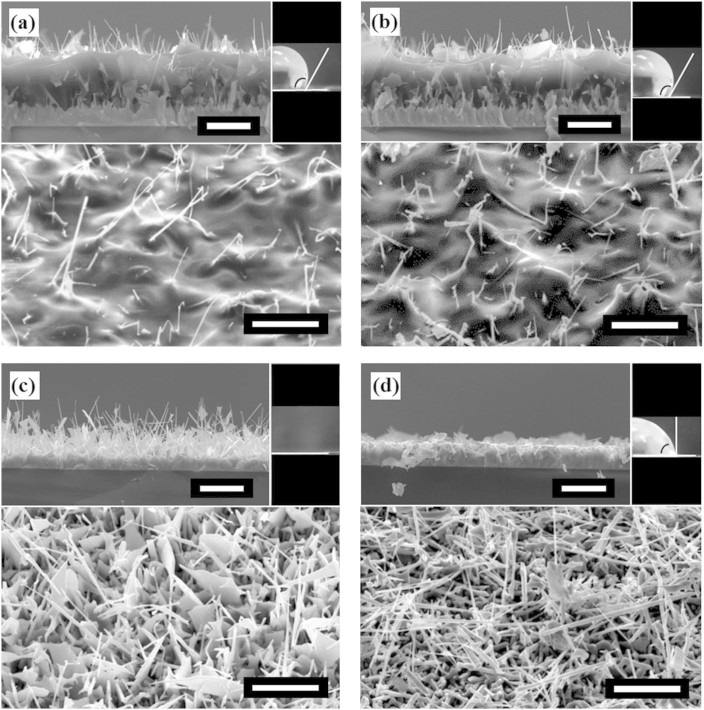
ZnO and Polymer Composite Surface. Scanning electron microscopy (SEM) images taken at 45° relative to the substrate normal (lower), cross sectional images (upper left) and water droplet on surface (upper right). (a) ZnO nanorods with polymer coating before wear. (b) ZnO nanorods with polymer coating after wear. (c) Uncoated ZnO nanorods before wear. (d) Uncoated ZnO nanorods after wear. The corresponding contact angles are (a) 120° (b) 118° (c) 0° and (d) 92°. White scale bars represent 10 μm.

As the initial set of results, we show that the surface nanoengineering has resulted in a hydrophobic surface. First, the contact angle of 120° before mechanical abrasion is much larger than the intrinsic value of 88° for the smooth polymer surface; Fig. 2(a). As an indication of the error of measurement, the standard deviation of five independent measurements of the contact angle is 4°. That is, increased hydrophobicity is achieved through the surface nanoengineering. Further, the contact angle changes only slightly to 118° after the mechanical abrasion test and remains within the error of measurement of the original value; Fig. 2(b). Both the morphology and the contact angle show that the surface is mechanically durable. The lack of morphological change before and after the mechanical abrasion test corresponds to the constancy of the contact angle. In passing, we note that the dynamic contact angle of the advancing surface is comparable to the static contact angle – 121° for the nanoengineered surface before abrasion and 124° after abrasion, and 87° for the smooth polymer surface; for the receding surface, the three values are 87°, 88°, and 63°. The change from 121° to 124° is within the error of measurement.

To appreciate the durability of the nanorods due to the polymer film, we demonstrate the fragility of the uncoated ZnO nanorods. Fig. 2(c) shows the uncoated ZnO nanorods before they undergo mechanical abrasion testing. The surface is superhydrophilic, and the high roughness results in a θ_s_ of 0°. After mechanical abrasion testing, the nanorods break and lie on the surface; Fig. 2(d). This causes the surface to become slightly hydrophobic, with a θ_s_ of 92°; the change of hydrophobicity may be related to the change in surface polarization, and the responsible mechanism is beyond the scope of this study. The change of hydrophobicity and surface morphology shows that ZnO nanorods alone are not mechanically durable. At the same time, the change of hydrophobicity also indicates that it is possible to control the hydrophobicity through variation of surface morphologies. In parallel, the polymer films are insensitive to wear and are mechanically durable. The intrinsic contact angle of the polymer surface is 88°, and does not change after abrasion; no physical change is observed after testing.

**Figure 3:**
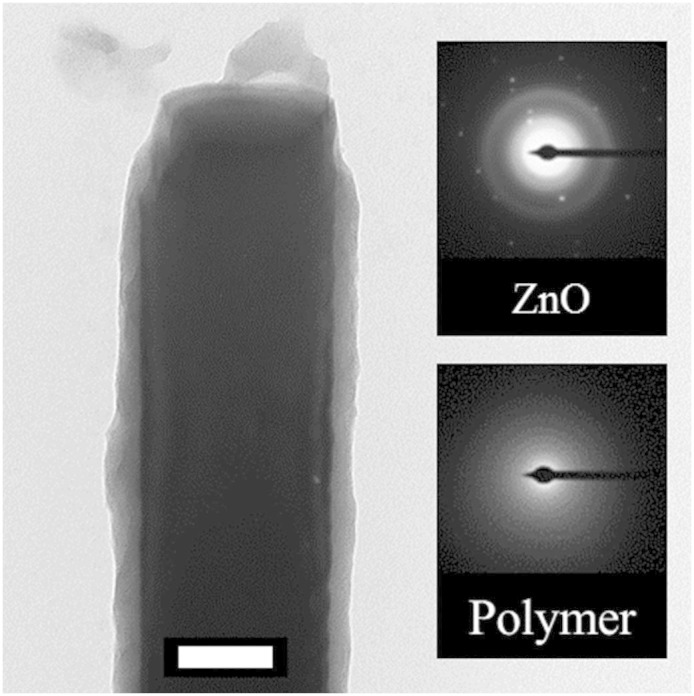
Core-shell nanorod. TEM image of an individual nanorod with electron diffraction patterns of the ZnO core and polymer shell as insets. White scale bar represents 0.1 μm.

To understand the lack of change of morphologies upon mechanical abrasion, we examine the structure of the nanorods. As shown in Fig. 3, the contrast of the bright field transmission electron microscopy (TEM) image reveals that the composite nanorod is in the form of a core-shell. The inset electron diffraction patterns show that the core is crystalline ZnO, and the shell is amorphous corresponding to the polymer. Indeed, this composite structure gives rise to mechanical strength and toughness, as we proposed at the beginning of this paper. Further, the cross-sectional scanning electron microscopy (SEM) images in Figs. 2(a) and (b) reveal a buffer layer between the exposed coated rods and the solid film of ZnO on the substrate. This buffer region, consisting of a bulk fill of polymer with ZnO nanorods, may provide additional mechanical durability do to its flexible support of the exposed nanorods. This added benefit is not the focus of this report and will not be further elaborated.

**Figure 4:**
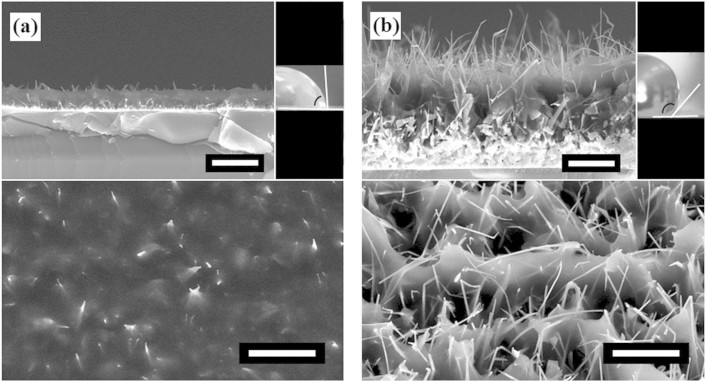
Controls of roughness and contact angle. SEM images taken at 45° relative to the substrate normal (lower), cross sectional images (upper left) and water droplet on surface (upper right). (a) A relatively smooth surface resulting in a low contact angle of 80° and (b) very rough surface resulting in high contact angle of 148°. White scale bars represent 10 μm.

To demonstrate control of the contact angle we change Δ. When Δ is very small, the resulting static contact angle of 80° is smaller than the intrinsic 88° of the polymer, affirming the Wenzel model; Fig. 4(a). When Δ is sufficiently large, the largest static contact angle we have achieved is 148°; Fig. 4(b). In Fig. 2(a) the rods are shorter and spaced farther apart, likely leading to water contact with the bulk surface in-between some nanorods, reducing the contact angle. It is critical to note that the optimization of hydrophobicity and that of mechanical durability are in competition. The larger Δ in Fig. 4(b) results in a larger contact angle than that in Fig. 2(a), but it also leads to less mechanical durability; after mechanical abrasion, the contact angle is reduced by 27° here in contrast to nearly no change in Fig. 2(b).

## Discussion and Summary

In passing, we will briefly discuss the cost and scalability of this method. First, while we chose ZnO nanorods fabricated using high temperature CVD as a prototype, ZnO nanorods of similar morphologies can also be fabricated through low-cost, chemical vapor deposition methods, or solution-phase synthesis methods, which are advantageous for manufacturing^17^,^18^. Additionally, aligned nanorods created by lithography or glancing angle physical vapor deposition could be used to provide better diameter and spacing control of the nanorods. For the application of the polymer, low cost spin coating is used. Alternatively, high throughput low cost methods such as spray deposition and jet printing are also viable alternatives.

To summarize, we have proposed and demonstrated the realization of combined hydrophobicity and mechanical durability through surface nanoengineering. By coating ZnO nanorods with polymer, we have achieved a surface that is hydrophobic with a contact angle of 120°. In contrast, the contact angle of polymer alone surface is 88°. The hydrophobicity derives from the nanoscale features - inherent from the nanorods - on the surfaces. More importantly, the coated surface is mechanically durable. This durability derives from the composite structure of the nanorods and the polymer. The combined hydrophobicity and mechanical durability may enable these engineered surfaces to be applicable in a range of technologies – such as protective coatings for buildings, or boat hulls.

## Methods

### Substrate preparation

We used {100} Si substrates, with a native oxide layer. The substrates are ultrasonically cleaned sequentially in acetone, methanol, then distilled water. A layer of gold (5 nm), to act as a catalyst layer, is deposited onto the wafers by electron beam physical vapor deposition at a rate of 0.02 nanometers per second in a vacuum of approximately 1 × 10^−3^ Pa. The rate is measured using a gold coated quartz crystal microbalance.

### Chemical vapor deposition of ZnO nanorods

The ZnO nanorods are grown by chemical vapor deposition (CVD) using the vapor liquid solid (VLS) method in a high temperature, low vacuum CVD reactor, following the method in Shim *et al.*^19^ An alumina boat containing 200 mesh zinc oxide powder (1 gram), is placed in the center of the quartz CVD tube. A second alumina boat containing the substrates is placed 20 cm downstream of flow in the tube. The center position of the tube reaches 1400°C, and the 20 cm downstream position remains at approximately 950°C during the gas flow stage. The reactor is evacuated to 1 Pa for several hours prior to heating to remove excess oxygen and moisture. After evacuation, the reactor is heated from room temperature to 1400°C at a rate of 10°C/min from room temperature to 700°C, 5°C/min from 700°C to 1000°C, and again at 10°C/min from 1000°C to 1400°C. Once the deposition temperature is reached, 99.998% argon (Ar) is then passed through the tube with a mass flow controller at a rate of 50 sccm and pressure is maintained at 27 kPa for 30 min. After 30 min, the Ar is shut off and low vacuum is resumed as the furnace is cooled at 5°C/min to room temperature.

### Polymer coating of ZnO nanorods

Immediately after the synthesis, we place the ZnO sample in a spin coater to coat the nanorods with polymer. The polymer used is Semi-Gloss Fast-Drying Polyurethane. Due to the proprietary nature of the polyurethane used, the exact molecular weight cannot be disclosed. A similar polyurethane has a polyol weight of 1180 Da with a functionality of 5.21^20^. The solvent is mineral spirits, the solid content is 50.15–50.90%, and the viscosity is 150–250 cps. The liquid polymer is dropped on the ZnO surface and spread across it evenly. The spin coater rotates between 500 and 4000 times per minute, over a variable amount of seconds to minutes that defines the thickness of polymer coating.

### Mechanical abrasion test

In the mechanical abrasion test, we pull the sample across 1000 grit sandpaper five times. The sample is placed face up on a low friction Polytetrafluoroethylene (PTFE) surface and attached to a weight of 105 grams that hangs freely over a low-friction pulley. This weight on the top of the stationary sand paper results in a pressure of 3.2 kPa. Local pressure at the point of contact between the nano-composite surface and sand paper “grit” may be higher due to the reduced contact area caused by the sandpaper roughness. The hanging weight is released and the sample slides under the stationary sandpaper.

### Characterization

Before and after each mechanical test, we characterize the surface morphology using a FEI Quanta 250 field emission gun (FEG) scanning electron microscope (SEM), and structure using a FEI Tecnai T12 transmission electron microscope (TEM). No metal coating, such as Au, is used for SEM imaging. For the measurement of contact angles, we use a RameHart Model 100 Goniometer with a droplet of distilled water of 5 μL placed gently on the surface. Dynamic contact angles are measured with a tilting cradle with the substrate 90° from the horizontal position.

## Author Contributions

The authors P.E., S.S., H.H., D.F., S.B. and T.F. were involved with designing the project. H.H., P.E. and S.S. analyzed the results, and wrote the manuscript together; P.E. performed the experiments. P.E., S.S., H.H., D.F., S.B. and T.F. collaborated during the discussion process and reviewed the manuscript.

## Figures and Tables

**Figure 1:**
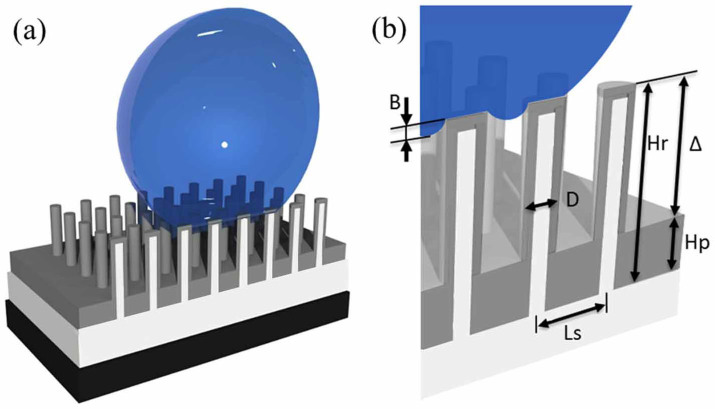
Schematic of nano-composite surface. (a) Schematic showing a silicon substrate (dark), ZnO film and nanorods (white), and polymer (gray); with a water droplet (blue). (b) Expanded view of the liquid-solid interface to clearly show nanorod diameter D, nanorod spacing Ls, nanorod height Hr, polymer height Hp, effective height Δ = Hr - Hp, and water droplet depth B. Not to scale.
